# Acute Retention of Urine—II

**Published:** 1908-07-11

**Authors:** 


### SURGERY.

### ACUTE RETENTION OF URINE—II.

The treatment of a case of acute retention of urine demands both patience and skill; patience because successive measures have often to be tried until one of them is eventually successful, and skill because the inefficient manipulation of a catheter may result in doing the patient some serious harm.

The first thing in all cases is to put the patient to bed. Urethral instrumentation should never be attempted on a patient who cannot be subsequently kept under observation, and this rule is equally important in all cases of stricture, whether acute retention has supervened or not. Not only is the passage of a catheter often associated with shock, but a condition known as "catheter fever" may be set up. The name of catheter fever is not a good one, since it implies a septic element; whereas, as a matter of fact, what is known as catheter fever may follow on the passage of an absolutely aseptic catheter on a patient with a stricture. Three varieties can be recognised of ascending severity: (1) characterised by a single rigor and a sudden rise of temperature within twelve hours of the passage of the instrument —this generally subsides spontaneously; (2) multiple rigors which persist for some time; and (3) suppression of urine without pyrexia, which may lead to delirium and even death. The exact method of causation of these phenomena is somewhat difficult to explain. It has been suggested that the instrumentation or even the anæsthetic, if one has been given, throws an extra demand on kidneys already in an unstable state, and so upsets their equilibrium. It is more probable, however, that the whole condition is a reflex one. The rapid onset of the symptoms is in favour of this, and so is the observed fact that a patient who has once suffered from such a condition is likely to do the same on each subsequent occasion.

The patient having therefore been put to bed, an attempt should be made to pass a catheter. A soft rubber catheter should be tried first, preferably a number 6 or 7. This will give information as to the site of the stricture, if it does nothing else. If the retention is largely due to spasm, and, as has been said, there is always a varying element of spasm in these cases, steady pressure even with a soft catheter may gradually overcome it. If not, a gum elastic catheter may be tried in a similar way. But it must be remembered that *no force must be used.* The catheter must be, so to speak, persuaded to go through the stricture. Force may not only do serious damage to the patient by creating a false passage, but will multiply the troubles of the surgeon because the presence of a false passage will make each subsequent attempt at instrumentation infinitely more difficult. If gentle manipulation with a gum elastic catheter fails, further attempts should not be indulged in until means have been taken to lessen the spasm. It is most important that the patient's bowels should be empty, and with this object an enema should be given. Opium should also be given by the mouth; the most satisfactory form in these cases is pulv. ipecac. co. gr. xv. Further the patient should be immersed in a bath as hot as he can bear. A temperature of 100° F. will be found as much as the ordinary patient can stand at first, but this can be subsequently increased if a trustworthy attendant is present in the bath-room.

The spasm is much relaxed by these measures, and in many cases the patient may pass some urine, even if only a little, in the bath; and it may be found possible to pass an instrument subsequently, where it was quite impossible before. Some authorities advocate an attempt to pass a silver catheter if both the soft rubber and the gum elastic ones have failed. But this is not really sound practice; as much force as it is ever advisable to use can be brought to bear with a gum elastic catheter, and a metal one is more likely to do harm than good, except in the hands of a specialist. This is especially true of the smaller sizes. A No. 1 silver catheter is literally one of the most dangerous instruments in surgery, and it is this which is responsible for a large percentage of the false passages one meets with. If a metal instrument is used, it should at any rate be one with a large bore.

But let us suppose that every means has been employed to overcome the spasm, and still no urine is passed, and no instrument can be introduced. If the patient's condition is extreme—that is, if the bladder is up to the umbilicus and he is in great agony, the bladder must be aspirated suprapubically. This is a small operation in itself, but it is nevertheless one which must be done with care and every possible precaution. The pubes must be shaved and made aseptic, and the trocar and cannula must be boiled. The trocar is inserted in the mid-line above the pubes with a slight inclination downwards. When the urine has been withdrawn the cannula may be removed and the small wound made covered over with gauze and collodion. The writer lays stress upon the necessity of proper asepsis in this operation, because he recently saw a case of fatal pelvic cellulitis which was set up by the introduction of a septic trocar through the unshaved pubes.

After the bladder has been emptied by suprapubic aspiration, the patient can sometimes pass his urine in the ordinary way, or if this is impossible a catheter or a filiform bougie can be introduced. If an instrument can be passed it should be tied in, at any rate for 24 hours. For some unexplained reason the presence of an instrument appears to cause the stricture to dilate, so that when it is removed an instrument two or three sizes larger can be introduced on the next day without much difficulty; after that time it is unnecessary to tie in the catheter. The stricture can be gradually enlarged by interrupted dilatation—an instrument one or even two sizes larger being passed daily, until a number 12 goes in with comparative ease. But if after trying all palliative measures, including suprapubic aspiration, no instrument can be passed, no alternative remains but to perform an external urethrotomy.

